# Predicting the Relationship Between Breastfeeding and Gross Motor Milestones Development: The Practice and Prevalence of Breastfeeding in Metropolitan Areas of Sindh, Pakistan

**DOI:** 10.7759/cureus.4039

**Published:** 2019-02-08

**Authors:** Anosh A Khan, Osama Mohiuddin, Iqra Wahid, Bareerah S Khan, Sulhera H Khan

**Affiliations:** 1 Internal Medicine, Dow University of Health Sciences, Karachi, PAK; 2 Medical Education and Simulation, Dow University of Health Sciences, Karachi, PAK

**Keywords:** lactation, breastfeeding, infants, mothers, education, socioeconomic status, motor milestones, pakistan

## Abstract

Objective

Breastfeeding is known to be beneficial for the health of both the child and the mother. The primary aim of this study is to assess the effect of lactation on the development of gross motor milestones. The evaluation of breastfeeding practices and the frequency of lactation among mothers living in the urban city of Karachi, Pakistan, is the secondary aim of this study so that interventions can be made to improve breastfeeding practices.

Methods

A cross-sectional study was conducted using a well-designed questionnaire. A population of 360 mothers living in Karachi, Pakistan, with children aged between two and six years, was selected. The questionnaire included demographic data, the duration of breastfeeding, the age of milestone development, and the educational and financial status of the mothers. The relationship between the duration of breastfeeding and the development of gross motor milestones was analyzed using the Pearson chi-square test via Statistical Package for the Social Sciences (SPSS) version 22.0. A p-value < 0.05 was rendered significant. Descriptive statistics were applied to calculate the frequency of the varying duration of breastfeeding among mothers with respect to their socioeconomic status and educational class.

Results

The study revealed that 68.6% of children were breastfed for four months or more with supplementary feed or solids started at four months or later (prolonged exclusive). Along with this, 6.4% were breastfed only before two months (short duration), 5.6% had been breastfed beyond two months but ceased before four months (intermediate duration) while 14.7% were breastfed for four months or more with supplementary feed or solids started before four months (prolonged partial). Mothers belonging to low (67.7%), moderate (67.5%), and higher (72.2%) socioeconomic status (SES) preferred to breastfeed for a prolonged exclusive duration. With respect to education, uneducated mothers (72.6%), mothers with primary education (63.6%), secondary education (65.90%), and tertiary education (68.6%) also breastfed for a prolonged exclusive duration. No statistically significant correlation was found between gross motor milestone development and the duration of breastfeeding (p-value > 0.05).

Conclusion

Breastfeeding was found to have an insignificant impact on the development of gross motor milestones despite the fact that mothers, irrespective of educational background and socioeconomic status, were found to be breastfeeding for a prolonged exclusive duration.

## Introduction

Mothers have been genetically imprinted with responsibility for their newborns, which starts with breastfeeding within a few hours of childbirth. Thereafter, breast milk remains the primary natural energy resource of infants, essentially providing more than half of their nutritional demand during the second half of the first year and up to one-third of their nutrition during the second year of life [1]. Breast milk contains the perfect amounts of calories and nutrients for the infant, especially during the first four to six months of life, providing the required velocity for growth [2]. It provides the right blend of benefits, encompassing the immunological, behavioral, and economic aspects while cementing mother-infant bonding [1].

Breastfeeding is the most effective method to ensure a child's health and sustenance. If done during the first six months of life, it can play a major role in preventing the death of over a million children each year [3]. According to the World Health Organization (WHO), infants should be exclusively breastfed for six months and then up to two years [4], making breastfeeding a preferable choice amongst mothers. Ample research has been done on the relation between the cognition of a child and breastfeeding, demonstrating a positive impact on development [5]. Several studies concluded a positive association between lactation and the emotional well-being and social and behavioral growth of the child [6]. It is also considered a prime marker of strengthening the mother-child bond [6] and a strong factor that predicts benefits to the mother's health [7].

Despite all the extensive studies that have examined the positive effects of breastfeeding, its impact on the gross motor development of the child still needs to be evaluated, especially in a third-world country like Pakistan where numerous families have children with small age gaps, making it grueling for mothers to meet the proper nutrient and caloric demands of each child. This highlights the relevance of why such a study needs to take place and thus our primary objective is to examine the relationship between the duration of breastfeeding and the development of gross motor milestones in children living in Karachi, Pakistan.

The positive effects of breastfeeding also depend upon its duration and frequency. Globally, less than 40% of infants under the age of six months are exclusively breastfed. For example, in the United States, 75% of mothers begin breastfeeding while only 43% of them continue until six months of age [3]. The graph of breastfeeding incidence in London, UK, indicates an increase from 62% in 1990 to 76% in 2005, to 81% by 2017 [3]. Although the duration of breastfeeding and the timing of weaning vary among different societies, it is mostly dependent upon the mother’s knowledge, choices, and cultural beliefs [1]. This indicates a necessity for a study to evaluate the pattern of breastfeeding practices in urban areas of Pakistan.

## Materials and methods

This was a cross-sectional study conducted on 360 mothers who were residents of Karachi and had mentally and physically healthy children between the ages of two and six years. Data were collected via preformed, pretested questionnaires, which were handed over to every consenting mother. The study was conducted from July 1, 2017, to July 1, 2018. The non-probability convenient sampling technique was used and the sample size was calculated using OpenEpi 3.03. Children who were under or above the aforementioned age range, had congenital deformities, an intellectual disability, a history of major accidents or trauma, and a family history of motor neuron disease were excluded from this study.

The questionnaire consisted of three sections. The first section covered the demographic data of the mother and child, including the birth weight of the child, birth order, gender of the baby, mother's age at the time of birth, mother's health issues, complications developed during delivery, and the mother's education and socio-economic status in terms of monthly income. The second section comprised information regarding the duration of breastfeeding and frequency of daily breastfeeding. The duration of breastfeeding was derived from the United Kingdom Infant Feeding guidelines [8]. The last section was based on questions regarding the age of achievement of six gross milestones using the WHO Multi-Centre Growth Reference Study [9].

The final data were analyzed via the Statistical Package for the Social Sciences (SPSS) version 22.0 (IBM, Armonk, NY, USA). The Pearson Chi-Square test was applied to assess the relationship between the duration of breastfeeding and the development of gross motor milestones. The frequencies of variation in breastfeeding duration among mothers, in terms of their educational and financial status, were also presented. Results were tabulated using Microsoft Word 2007 (Microsoft Corporation, Washington, US). Percentages and frequencies were calculated for qualitative and quantitative variables by descriptive statistics for the mother's health issues during childbirth, educational background, socioeconomic status, etc. A p-value of < 0.05 was considered significant.

## Results

Table 1 shows that out of 360 participants, 48% had a birth weight between 3.0 kg and 3.9 kg. With respect to birth order, 31.1% were the second child while less than a percent were the eighth child. Males and females were in an approximately equal ratio. At the time of birth, about one-third of the mothers were aged between 21 and 25 years. The majority of mothers had no health complications during pregnancy and delivery. The stratification of mothers in terms of educational background and socioeconomic status is also presented below.

**Table 1:** Demographics and Sample Characteristics PKR = Pakistani Rupee

Characteristics	Classes	N (Frequency)	% (Percentage)
Birth Weight (kg)			
	1kg – 1.9 kg	19	5.2
	2kg – 2.9kg	146	40.5
	3kg – 3.9kg	173	48
	4kg – 4.9kg	22	6.1
Birth Order			
	1	105	29.2
	2	112	31.1
	3	76	21.1
	4	33	9.2
	5	18	5.0
	6	9	2.5
	7	4	1.1
	8	3	0.83
Gender of the Child			
	Male	179	4.7
	Female	181	50.3
Mother’s Age at the Time of Birth (years)			
	15 - 20	44	12.2
	21 – 25	117	32.5
	26 – 30	98	27.2
	31 – 35	48	13.3
	36 – 40	49	13.6
	41 – 45	3	0.83
	45 - 50	1	0.27
Health Issues During Child Birth			
	None	259	71.9
	Hypertension	28	7.8
	Diabetes	8	2.2
	Postpartum Depression	19	5.3
	Anemia	37	10.3
	Any Other	9	2.5
Monthly Income (PKR) Socioeconomic Status (SES)			
	< Rs.50,000 (Low)	158	43.9
	Rs. 50,000 – Rs.100,000 (Moderate)	123	34.2
	>Rs. 100,000 (High)	79	21.9
Mother’s Education			
	Uneducated	95	26.4
	Primary Education	15	15.3
	Secondary Education	44	12.2
	Tertiary Education	166	46.1

In terms of duration of breastfeeding, 68.6% of the kids were breastfed for a prolonged exclusive period, 14.7% were breastfed for a prolonged partial period, 6.4% were breastfed for a short duration, 5.6% were breastfed for an intermediate duration, and 4.75% were never breastfed at all. Nearly half of the children were fed on demand. The frequency and duration of breastfeeding are presented in Table 2.

**Table 2:** Frequency and Duration of Breastfeeding

Characteristics	Classes	N (Frequency)	%(Percentage)
Frequency of feeding the child			
	Every 1 – 2 hours	63	17.5
	Every 2 – 3 hours	76	21.1
	Every 3 – 4 hours	36	10.0
	On demand	185	51.4
Duration of breastfeeding			
	Never Breastfed	17	4.7
	Short Duration (ceased before 2 months of age)	23	6.4
	Intermediate Duration (ceased breastfeeding after 2 months but before 4 months)	20	5.6
	Prolonged Partial (breastfed for 4 months or more with supplementary feed or solid started before 4 months)	53	14.7
	Prolonged Exclusive (breastfed for 4 months or more with supplementary feed or solid started at 4 months or later)	247	68.6

Table 3 reveals the details of the ages of development of six major gross milestones with respect to frequency and percentage of children.

**Table 3:** Frequency of Age of Achievement of Gross Motor Milestones

Characteristics	Classes	N (Frequency)	% (Percentage)
Age of sitting without support (months)			
	3.5 – 4	12	3.3
	4 – 4.5	32	8.9
	4.5 – 5	58	16.1
	5.5 – 6	2	0.6
	6– 6.5	67	18.6
	> 6.5	64	17.8
Age of standing with assistance (months) :			
	< 5	7	1.9
	5 - 6	16	4.4
	6 – 7	40	11.1
	7 - 8	86	23.9
	8 – 9	112	31.1
	> 9	99	27.5
Age of hands and knee crawling (months) :			
	< 6	39	10.8
	6 – 7	84	23.3
	7 – 8	105	29.2
	8 – 9	68	18.9
	9 – 10	43	11.9
	10 – 11	11	3.1
	> 11	10	2.8
Age of walking with assistance (months) :			
	< 9	56	15.6
	9 – 10	87	24.2
	10 – 11	87	24.2
	11 – 12	71	19.7
	12 – 13	36	10.0
	> 13	21	5.8
Age of standing alone (months) :			
	<9	20	5.6
	9 – 10	80	22.2
	10 – 11	67	18.6
	11 – 12	91	25.3
	12 – 13	61	16.9
	> 13	41	11.4
Age of walking alone (months) :			
	< 10	23	6.4
	10 – 11	39	10.8
	11 – 12	78	21.7
	12 – 13	77	21.4
	13 – 14	74	20.6
	>14	69	19.2

**Figure 1:**
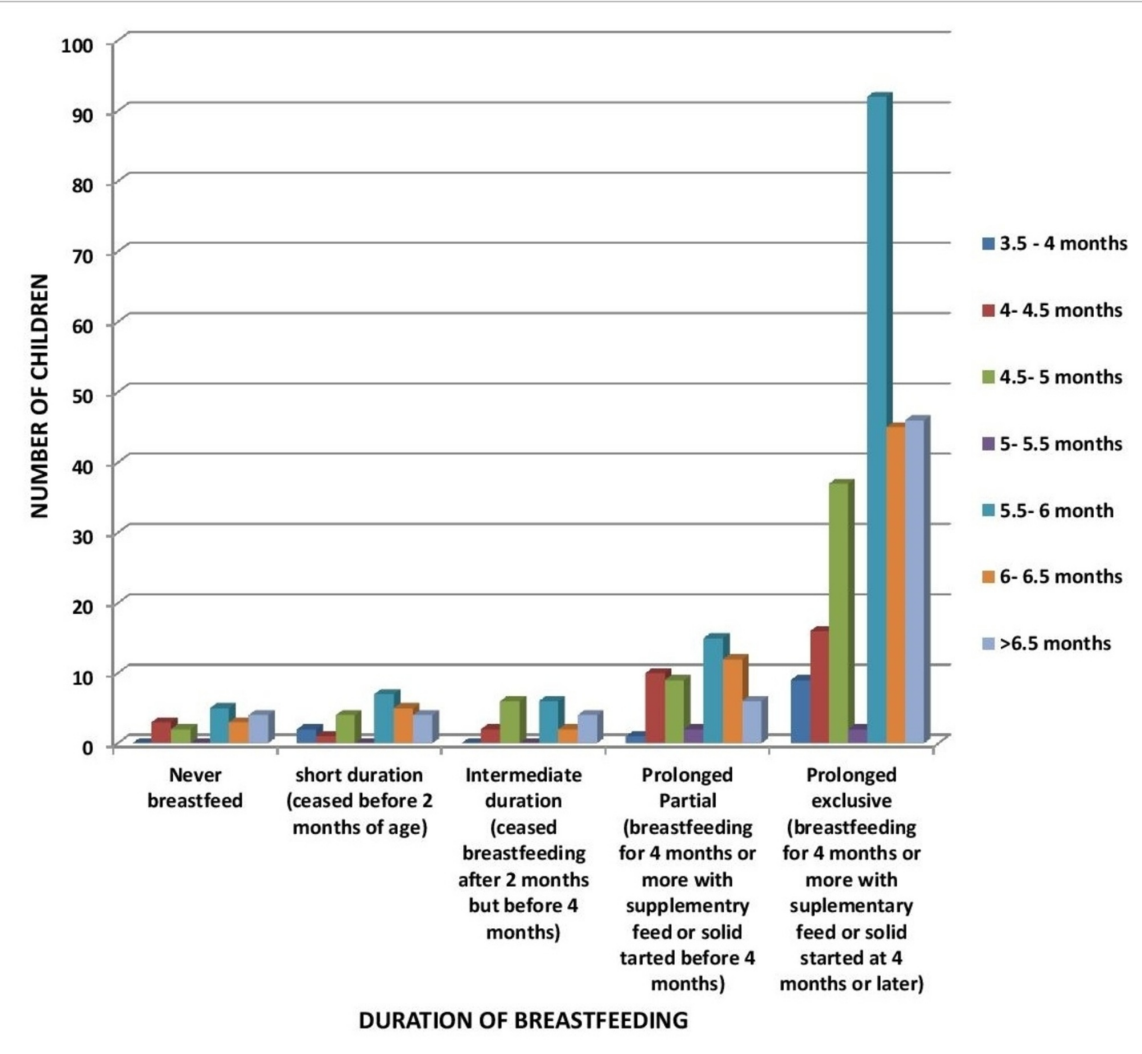
Relation of Breastfeeding to Age of Sitting Without Support

**Figure 2:**
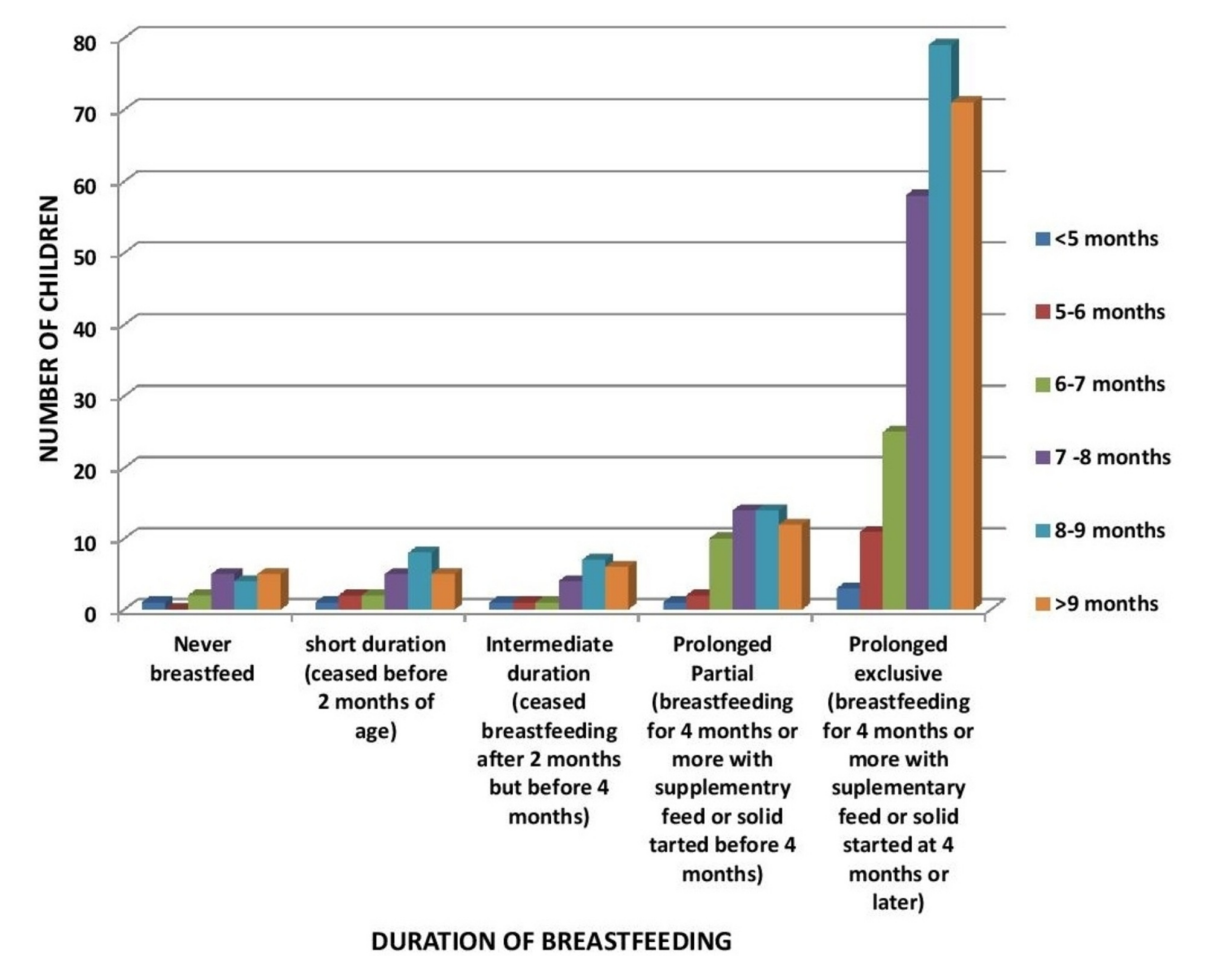
Relation of Duration of Breastfeeding to Age of Standing with Assistance

**Figure 3:**
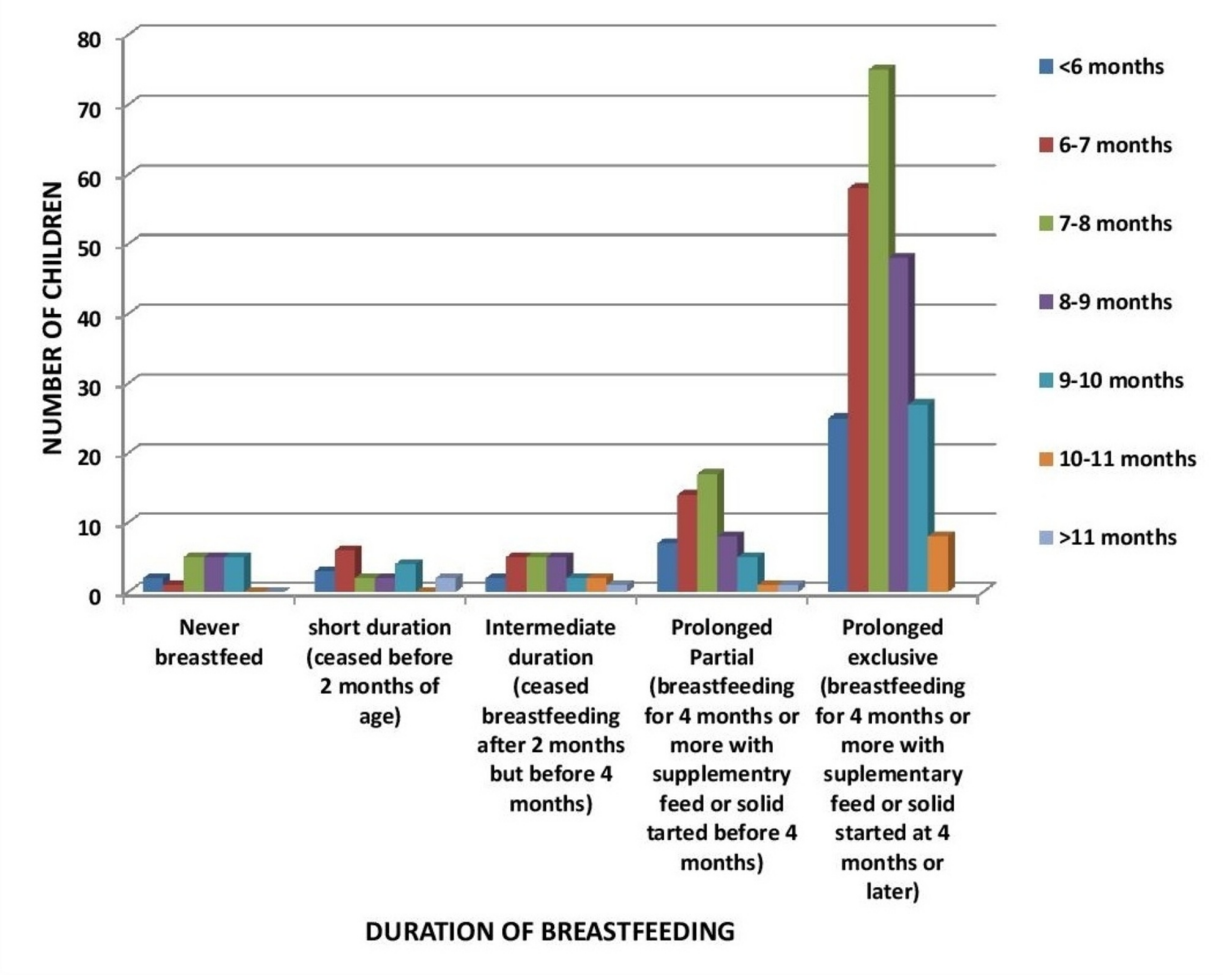
Relation of Duration of Breastfeeding to Age of Hand and Knee Crawling

**Figure 4:**
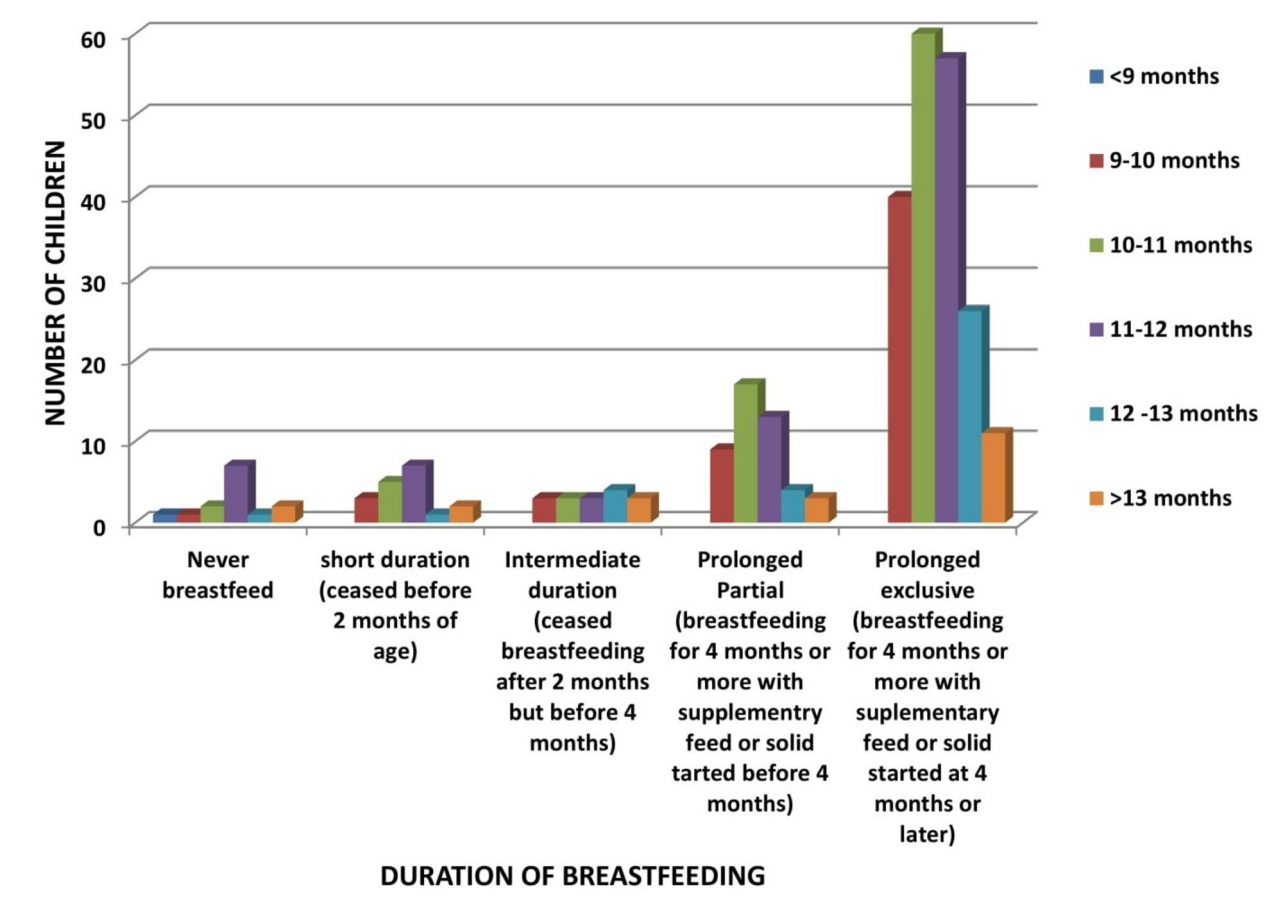
Relation of Duration of Breastfeeding to Age of Walking with Assistance

**Figure 5:**
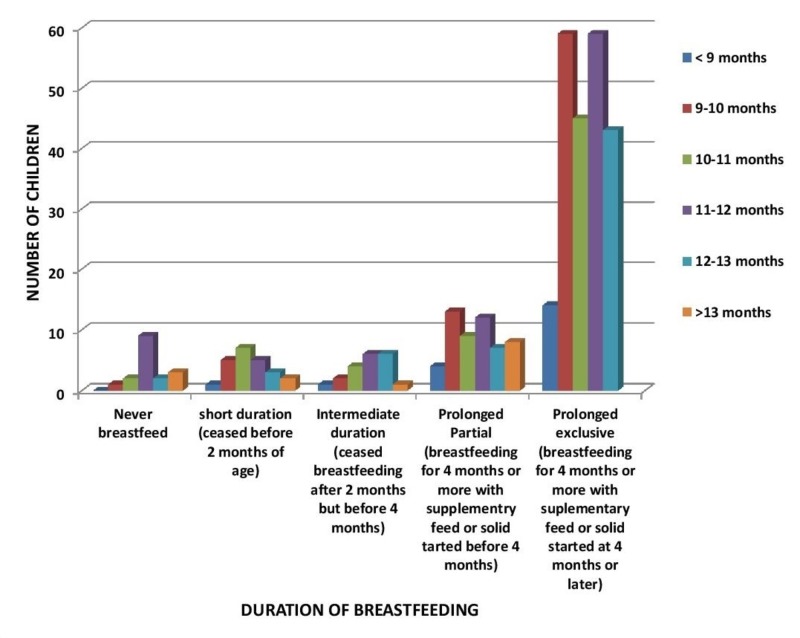
Relation of Duration of Breastfeeding to Age of Standing Alone

**Figure 6:**
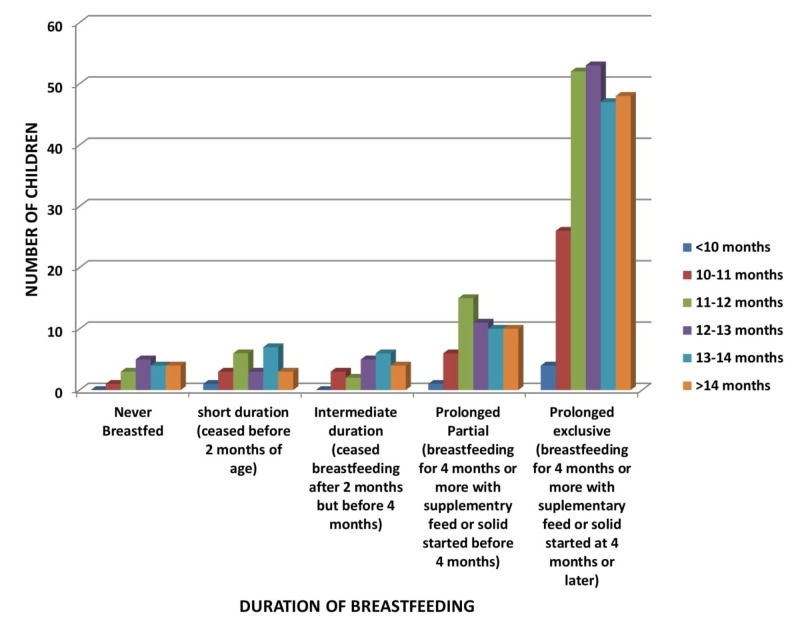
Relation of Duration of Breastfeeding to Age of Walking Alone

A statistically insignificant correlation (p-value > 0.05) was observed between the duration of breastfeeding and the age of development of gross milestones. This is exhibited by Figures 1-6. In Figure 1, the majority of the children were breastfed for a prolonged exclusive duration, among which 91 children (majority) were sitting without support at 5.5 months to six months (p-value 0.606).

In Figure 2, the majority of the children were breastfed for a prolonged exclusive duration, among which 78 (the majority) were standing with assistance at eight to nine months (p-value 0.92).

In Figure 3, the majority of the children were breastfed for a prolonged exclusive duration, among which 74 children (the majority) were crawling by seven to eight months (p-value 0.569).

In Figure 4, the majority of the children were breastfed for a prolonged exclusive duration, among which 59 children (majority) were walking with assistance by 10 to 11 months (p-value 0.612).

In Figure 5, the majority of the children were breastfed for a prolonged exclusive duration, among which 58 children were standing alone by nine to 10 months.

In Figure 6, the majority of the children were breastfed for a prolonged exclusive duration, among which 52 children (the majority) were walking alone by 12 to 13 months (p-value 0.836).

In Table 4, the majority of mothers with no education or primary, secondary, or tertiary education breastfed their children for a prolonged exclusive duration. Details of the duration of breastfeeding with respect to the educational status of the mothers are shown below.

**Table 4:** Frequency of Breastfeeding Duration on the Basis of Educational Background of Mothers

Duration of breastfeeding	Educational status of mother (%)			
	Uneducated	Primary Education	Secondary Education	Tertiary Education
Never Breastfed	8.42	5.45	2.27	3.01
Short Duration (ceased before 2 months of age)	6.31	7.27	9.09	5.42
Intermediate Duration (ceased breastfeeding after 2 months but before 4 months)	2.10	9.09	9.09	5.42
Prolonged Partial (Breastfed for 4 months or more with supplementary feed or solid started before 4 months)	10.52	14.54	13.63	17.46
Prolonged Exclusive (breastfed for 4 months or more with supplementary feed or solid started at 4 months or later)	72.60	63.63	65.90	68.67

In terms of socioeconomic (SES) status, the majority of mothers belonging to low (67.72%), moderate (67.47%), and high SES (72.15%) lactated their children for a prolonged exclusive period. Less than one-tenth of mothers belonging to each SES were those who never breastfed at all. Further details are elaborated in Table 5.

**Table 5:** Frequency of Duration of Breastfeeding on the Basis of Socioeconomic Status (SES) of Mothers

Duration of breastfeeding	Socioeconomic status (SES) of mothers (%)		
	Low SES	Moderate SES	High SES
Never Breastfed	6.96	2.43	3.79
Short Duration (ceased before 2 months of age)	8.22	4.06	6.32
Intermediate Duration (ceased breastfeeding after 2 months but before 4 months)	4.43	8.13	3.79
Prolonged Partial (Breastfed for 4 months or more with supplementary feed or solid started before 4 months)	12.65	17.88	13.92
Prolonged Exclusive (breastfed for 4 months or more with supplementary feed or solid started at 4 months or later)	67.72	67.47	72.15

## Discussion

This study was primarily aimed at investigating the association between the duration of breastfeeding and the development of six major gross motor milestones. According to our study, the correlation stands to be insignificant (p-value > 0.05). A study conducted on Honduran infants compared the age of achievement of several milestones and concluded that infants who were exclusively breastfed tended to crawl earlier, with a significant correlation between them. However, the rest of the milestones do not have a significant association with exclusive breastfeeding [10]. Another study held that the addition of solid food alongside breastfeeding was associated with a relatively faster time to stand and walk [11]. The introduction of solid food provides nutrients and calories that help prevent developmental delays and stunting along with nutritional imbalance and childhood illness [1]. Likewise, Siregar et al. [12] also identified an insignificant association between breastfeeding duration and gross motor development. Even after adjusting for confounding factors, a study conducted in Greece also had similar results. However, a positive correlation was seen between breastfeeding duration and fine motor skills, a factor not observed in this study [5].

Despite all these results, breast milk does have some role in gross motor milestone development, which lies in its biochemical composition. It contains ample quantities of long-chain polyunsaturated fatty acid (LC-PUFAs), which is an essential component of grey and white matter. High levels of these were found in the cerebellum of breastfed infants, which helped them attain motor balance, control, and posture maintenance early on [13]. Some researches concluded that there is less likelihood of gross motor delay with increased duration of breastfeeding such as one conducted in the United Kingdom on 14,000 infants [8]. This contradiction is probably due to the small sample size of this study, a major drawback. Kusuma et al. [14] elaborated that motor development is directly linked to the nutritional status, which, in turn, depends on the duration of breastfeeding, mother's education, and birth weight, proving that there are complex interconnections of multiple factors that contribute to child motor development. Maternal education is one such factor. We concluded that out of 360 mothers under observation, only 46.1% of the mothers had tertiary education followed by 26.38% of mothers having no education, as seen in Table 4. More than half of the mothers of each educational background were breastfeeding for a prolonged exclusive period and, in fact, mothers with tertiary education breastfeeding for a prolonged exclusive duration were about 4% less than mothers with no education and a prolonged exclusive duration of breastfeeding. Educated mothers had jobs and inconsistent maternity leave that affected the breastfeeding duration, as also stated in a meta-analysis [15]. One of the reasons is the mother’s hesitancy and embarrassment to feed in public or at their workplace, in addition to the belief that babies cannot be easily weaned if they become habituated to breast milk [16]. Contrarily, some studies concluded that educated mothers had higher odds of exclusive breastfeeding [17], claiming that education provides access to more media channels through the Internet, posters, or family health cards [18].

Like education, financial status also impacts breastfeeding duration. As seen in Table 5, the majority (43.8%) of mothers belonged to low SES and a higher percentage with high SES breastfed for a prolonged exclusive duration. This is probably because mothers with higher SES do not find it necessary to work outside and can stay at home and breastfeed for long periods [19]. Studies conducted in Ethiopia [18,20] also reinforced the same finding. It might be due to the fact that mothers belonging to higher SES are at more financial ease to seek maternal health care advice from different lactation counselors and health care providers who can advise them about the benefits of prolonged breastfeeding [18]. A study conducted in Haiti [21] suggested that widespread poverty leads to a nutrient deficiency in mothers, resulting in physical weakness and a perception of poor milk production, eventually prompting them to start a complementary feed early onwards. Most households in Pakistan have a trend of multiple children, especially in its most urban city of Karachi, where families belonging to low SES migrate from different provinces in search of employment. The concept of large families originates among these families from the belief that children can support the family unit. As a result, there is less of an age gap among the children, and mothers are not able to breastfeed them for a longer duration.

There are some strong features of this study. First, it is unbiased due to a random selection of mothers. Second, it elaborates the duration of lactation extensively on a monthly basis, instead of using limited variables like "no breastfeeding" and "breastfeeding." Third, SES and educational background are also categorized into detailed groups to create a better picture of reality. Fourth, several studies only discussed generalized motor development while we evaluated the development of gross motor milestones. Lastly, such a focused study has not been done recently in Pakistan, which again highlights the significance of this study.

Nevertheless, there were limitations in the research conducted too. First, there is a lack of adjustment of confounding factors, such as maternal health at the time of breastfeeding, weaning of solid food, and the introduction of formula milk, which could be some of the causes for the insignificant correlation that was found. Second, our sample size was relatively small. Larger sample size could have given a better idea about the main findings. Third, as mothers of older children were required to recall the age of certain milestone achievements, this could have caused a recall bias.

## Conclusions

We have concluded that lactation has an insignificant association with the development of gross motor skills despite the fact that kids who were lactated for a long time did achieve gross milestones at a relatively early age. However, further extensive study is required to study the confounders that come into play. Prolonged breastfeeding is most prevalent among uneducated mothers and those belonging to high SES but still not close to 100%. Efforts should be to put into making health care access and lactation counseling more approachable in terms of finance, media channels, and easy, comprehensive language. New policies should be made to give extended maternal leave so that educated working mothers can also breastfeed for a long duration. These measures can help promote the practice of breastfeeding for a longer duration (as recommended by WHO) to every Pakistani mother.
